# Long‐term disease control after upfront chemotherapy and surgery in a patient with primary prostate leiomyosarcoma

**DOI:** 10.1002/iju5.12400

**Published:** 2021-11-30

**Authors:** Toshiaki Kawaguchi, Toshikazu Tanaka, Masaru Ogasawara, Iwabuchi Ikuya

**Affiliations:** ^1^ Department of Urology Aomori Prefectural Central Hospital Aomori‐shi Aomori Japan

**Keywords:** aggressive neoplasm, MAID regimen, preoperative chemotherapy, prostate leiomyosarcoma, total pelvic exenteration

## Abstract

**Introduction:**

Prostate leiomyosarcoma is a rare, aggressive neoplasm.

**Case Presentation:**

A 52‐year‐old man presented with worsening frequent micturition and painful urination. Rectal examination revealed a significantly enlarged prostate. Magnetic resonance imaging showed a large prostate tumor with urinary bladder and bilateral seminal vesicle invasion. A prostate biopsy revealed diffuse proliferation of pleomorphic atypical cells. Immunohistochemistry confirmed the diagnosis of prostrate leiomyosarcoma. The patient received three cycles of the mesna, doxorubicin, ifosfamide, and dacarbazine regime (mesna 6000 mg/m^2^, doxorubicin 60 mg/m^2^, ifosfamide 7500 mg/m^2^, and dacarbazine 900 mg/m^2^) at 4‐week intervals. The tumor shrank by 28% and exhibited necrotic changes. He underwent total pelvic exenteration with en bloc resection of the prostate, bladder, rectum, and anus. Pathological surgical margin was negative. The patient is alive with no disease at 5 years postoperatively.

**Conclusion:**

Neoadjuvant chemotherapy and surgical resection are essential to achieve a long‐term survival of patients with localized prostate leiomyosarcoma.

**Abbreviations & Acronyms**
α‐SMA: α‐smooth muscle actin
CI: confidence interval
CT: computed tomography
H&E: hematoxylin and eosin
LMSOP: primary leiomyosarcoma of the prostate
MAID: mesna, doxorubicin, ifosfamide, and dacarbazine
MRI: magnetic resonance imaging
OS: overall survival
PSA: prostate specific antigen

**Keynote message**

Prostate leiomyosarcoma is an aggressive neoplasm. Complete resection with negative margins is essential, but surgery alone cannot improve its prognosis. The MAID (mesna, doxorubicin, ifosfamide, and dacarbazine) regimen administered as preoperative chemotherapy is effective.

## Introduction

Sarcomas of the prostate are rare and account for 0.1–0.2% of all malignant prostatic tumors. Rhabdomyosarcomas of prostate are known as the most frequent tumors in childhood, whereas leiomyosarcomas are the most common sarcomas involving the prostate in adults.^1^ Prostate sarcomas have conventionally been treated with radical surgery including prostatectomy, cystoprostatectomy, and total pelvic exenteration. The prognosis of prostate sarcomas has been poor with surgery alone. We report the case of a patient with prostate leiomyosarcoma treated with total pelvic exenteration after neoadjuvant chemotherapy.

## Case report

**Fig. 1:**
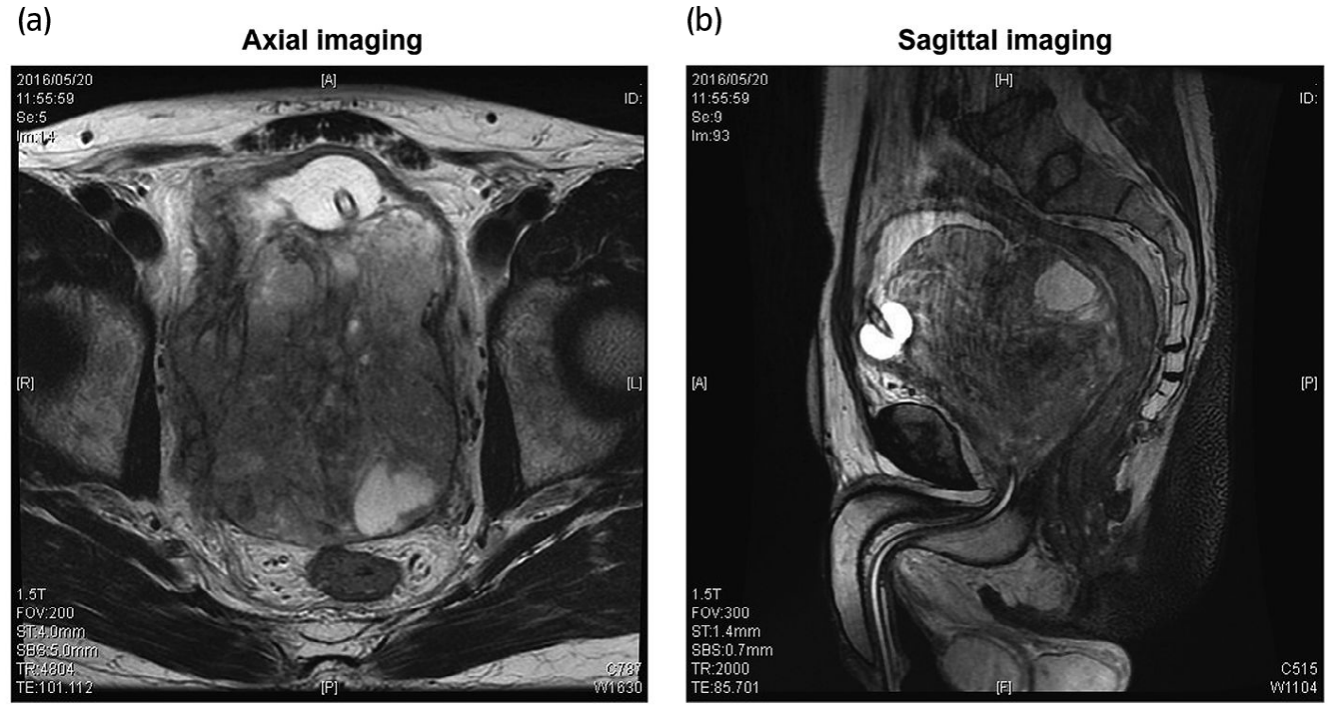
MRI (T2‐weighted image). (a) Axial view, (b) Sagittal view. MRI showed a whole enlargement of prostate tumor (10.6 × 9.3 cm) with heterogeneous signals. The prostate tumor protruded into the urinary bladder and invaded the bilateral seminal vesicles, but the rectal wall and pelvic lateral walls were not invaded.

**Fig. 2:**
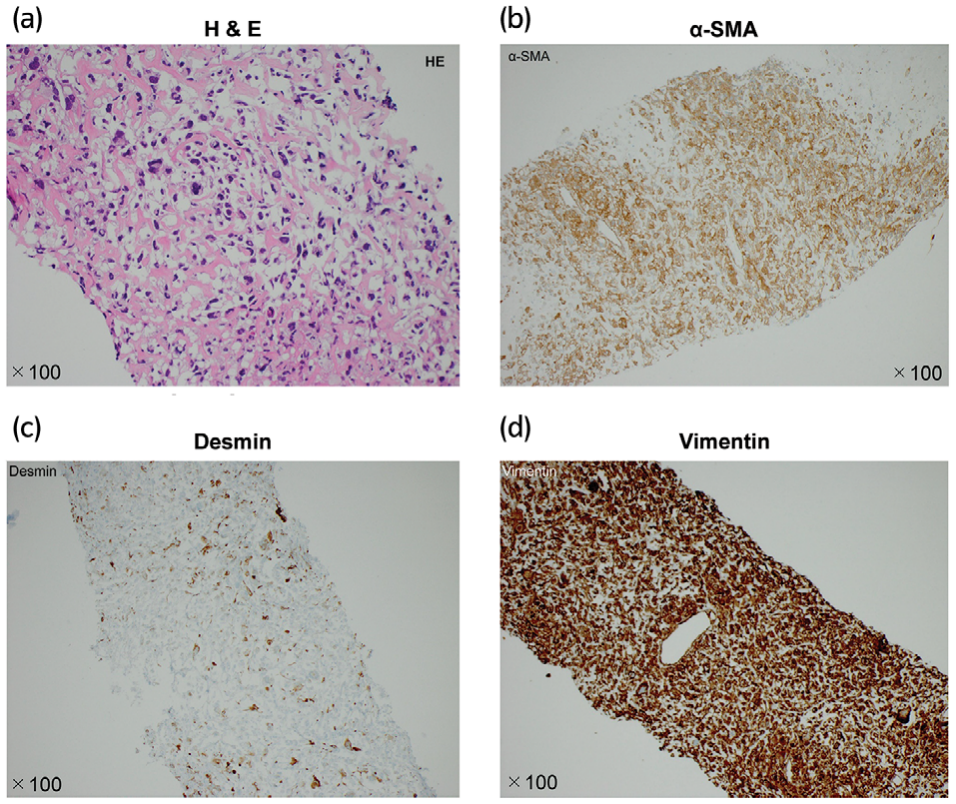
Microscopic findings from the prostate biopsy. (a) H&E staining shows mainly diffuse proliferation of atypical cells with small, hyperchromatin nuclei, and pleomorphic, polynuclear gigantic cells. Immunohistochemical analysis shows staining for (b) α‐SMA, (c) desmin, and (d) vimentin in tumor cells.

A 52‐year‐old man presented with a complaint of frequency and difficulty in urination since February 2016. The patient visited a urologic clinic and received antibiotics and an α1‐blocking agent. However, the perineal pain and difficulty in urination worsened. The patient was referred to our hospital in May 2016. Rectal examination revealed a significant enlargement of the prostate with unclear margins. The prostate specific antigen level was 0.8 ng/mL. MRI (T2 weighted image) showed a heterogeneous, large, prostate tumor (10.6 × 9.3 cm) with invasion into the urinary bladder and bilateral seminal vesicles without invasion of the rectal wall or internal obturator muscle, as shown in Figure 1. CT demonstrated right hydronephrosis due to tumor compression but no metastasis to lymph nodes or visceral organs. A transrectal ultrasound‐guided prostate biopsy was performed and the results are shown in Figure 2. H&E‐stained sections revealed diffuse proliferation of pleomorphic atypical cells with chromatin‐rich small nuclei. Leiomyosarcoma of the prostate was confirmed via immunohistochemical staining; tumor cells were positive for α‐SMA, desmin, and vimentin and negative for androgen receptor, NKK3.1.

**Fig. 3:**
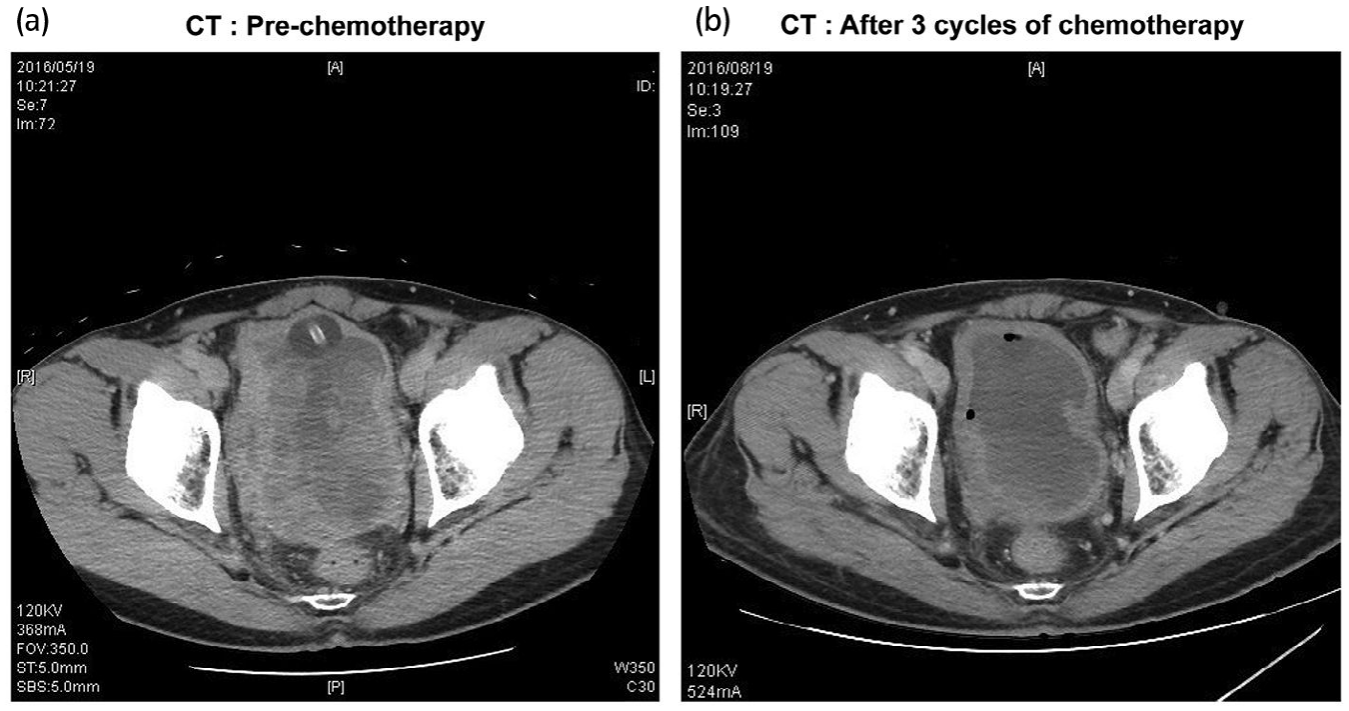
CT images (a) prechemotherapy and (b) after three cycles of neoadjuvant chemotherapy. After the three cycles of MAID regimen, the tumor shrank by 28% and necrotic changes were obvious.

**Fig. 4:**
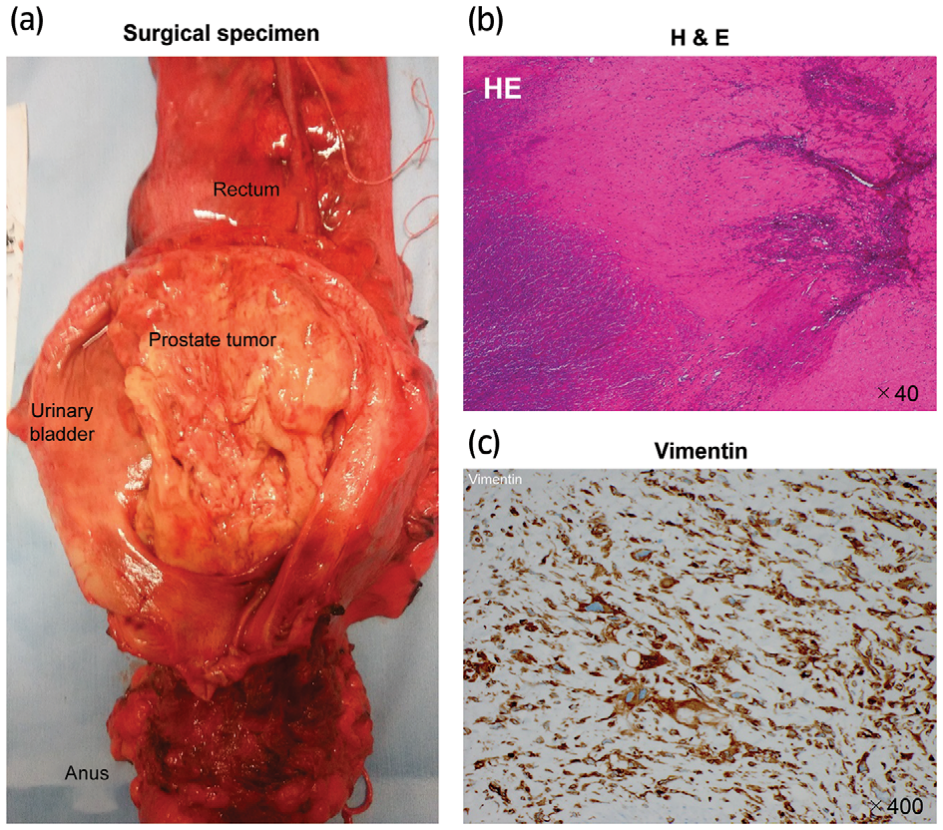
Postsurgical macroscopic and microscopic findings. (a) Macroscopic findings. The prostate, urinary bladder, and rectum (with anus) were completely resected. (b) H&E staining. Prostate tumor cells were mostly necrotic and interstitial vitrification was obvious. Only a few tumor cells remained. (c) Immunohistochemistry for vimentin showed scattered viable cells in the bladder muscle.

After consulting the oncologist at our hospital, we decided to apply preoperative chemotherapy with MAID regime. The patient underwent three cycles of MAID regime comprising mesna 6000 mg/m^2^, doxorubicin 60 mg/m^2^, ifosfamide 7500 mg/m^2^, and dacarbazine 900 mg/m^2^, every 4 weeks from June 2016. Grade 3 febrile neutropenia was observed after every cycle, despite subcutaneous pegfilgrastim injections. CT after three cycles of chemotherapy showed 28% tumor shrinkage and obvious necrotic changes in the tumor, as shown in Figure 3. Finally, total pelvic exenteration was performed, wherein the prostate, bladder, rectum, and anus were en bloc resected and replaced with an ileal conduit and sigmoid colostomy in September 2016. The surgical pathology is shown in Figure 4. The H&E‐stained sections revealed that nearly all of the tumor cells were necrotic and only those invading the muscle layer of the urinary bladder were viable. The surgical margins were microscopically negative. The patient received no adjuvant chemotherapy and is alive with no disease at 5 years postoperatively.

## Discussion

LMSOP is very rare, and <200 cases have been reported to date.^2^ Most common symptoms of LMSOP are urinary obstruction and urinary frequency, such as urgency, and nocturia. As the tumor grows, hematuria, perineal and/or rectal pain, constipation, burning on ejaculation and weight loss are observed. LMSOP presents as nonspecific enlargement of the prostate upon physical examination and diagnostic imaging. Serum PSA levels are not almost higher than within normal limits; therefore, most patients are diagnosed by using ultrasound‐guided needle biopsy.^3^ Microscopic findings of LMSOP are characterized by high‐grade hypercellular lesions comprising eosinophilic spindle‐shaped cells with mitotic changes, prominent necrotic lesions, and cystic degeneration. Vimentin, SMA, and desmin are commonly expressed in neoplastic cells and cytokeratin in approximately 25% of the cases.^3^

Clinical outcomes for patients with LMSOP are poor. A review of 34 patients with LMSOP demonstrated that median survival is 17 months (95% CI 20.7–43.7 months) and the survival rates at 1,3,5 years are 68%, 34%, and 26%, respectively. The OS of patients with metastasis at initial diagnosis was worse than that of those with no metastasis (median OS, 5 *vs* 20 months, *P* = 0.018). The OS of patients with incomplete resection was worse than that of those showing negative surgical margin (median OS, 13 *vs* 41 months, *P* = 0.008).^3^ For patients with localized disease, complete resection with negative surgical margin is the optimal treatment and represents the possibility of curing disease. However, Wang *et al*. reported there was no significant correlation between the status of surgical margin and OS.^4^

Sexton *et al*.^5^ and Musser *et al*.^6^ reported their experiences with prostate sarcoma in 21 (12 with LMSOP) and 38 patients (13 with LMSOP), respectively. As the prognosis of patients with prostate sarcoma who underwent only surgery was poor, they proposed that multimodal therapy should be performed. Of the six patients with stage Ⅲ prostate sarcoma treated with preoperative chemotherapy and/or radiation therapy, four survived and were with disease‐free at mean time of 70 months.^5^ Agents used for neoadjuvant chemotherapy included combination of gemcitabine and docetaxel; doxorubicin and ifosfamide; or cyclophosphamide, vincristine, and doxorubicin.^6^ Recently, Ball *et al*.^7^ reported that preoperative chemoradiation therapy for eight patients (three with LMSOP) with stage Ⅲ, high‐grade prostate sarcoma improved OS. Neoadjuvant radiotherapy was administered at 45–50.4 Gy. The MAI (mesna, doxorubicin, and ifosfamide) was administered as concurrent chemotherapy. The median OS was 67.8 months, and the survival rate at 1,3,5 years were 100%, 55.6%, and 55.6%, respectively. All three LMSOP patients were survived.

Ogura *et al*.^8^ reported that perioperative chemotherapy with a modified MAID regimen resulted in a favorable outcome for patients with high‐grade non‐small round cell soft sarcomas. Among the 26 patients evaluated, 8 achieved a partial response, 15 had stable disease, and 3 patients showed disease progression. The 5‐year OS and disease‐free survival rates were 86% and 77%, respectively. Grade 3/4 hematopoietic toxicity, especially neutropenia (95%) was frequently observed, but no other serious adverse events were noted. Therefore, we concluded that the MAID regimen is one of the most appropriate neoadjuvant chemotherapy for patients with LMSOP.

## Conclusion

A multimodal approach, including systemic chemotherapy and radical surgery, should be considered to achieve a long‐term survival of patients with localized prostate leiomyosarcoma.

## Author contributions

**Toshiaki Kawaguchi:** Conceptualization; data curation; formal analysis; funding acquisition; investigation; methodology; project administration; resources; software; validation; visualization; writing‐original draft; writing‐review & editing. **Toshikazu Tanaka:** Data curation; resources; software; validation; visualization. **Masaru Ogasawara:** Data curation; project administration; resources; supervision; validation. **Iwabuchi Ikuya:** Funding acquisition; methodology; project administration; supervision; validation.

## Conflict of interest

The authors declare no conflict of interest.

## Approval of the research protocol by an Institutional Reviewer Board

Not applicable.

## Informed consent

Not applicable.

## Registry and the Registration No. of the study/trial

Not applicable.
